# Intra-Individual Differences of the Femoral Cortical Thickness Index in Elderly Patients with a Proximal Femoral Fracture

**DOI:** 10.3390/jcm14082654

**Published:** 2025-04-12

**Authors:** Flurina Guyan, Manuel Waltenspül, Michael Dietrich, Method Kabelitz

**Affiliations:** 1 Medical School, University of Zürich, 8006 Zürich, Switzerland; flurina.guyan@uzh.ch; 2 Clinic for Orthopaedics, Hand Surgery and Trauma Surgery, Stadtspital Zürich, Tièchestrasse 99, 8037 Zürich, Switzerland; manuel.waltenspuel@stadtspital.ch (M.W.); michael.dietrich@stadtspital.ch (M.D.)

**Keywords:** cortical thickness index, osteoporosis, proximal femoral fracture, geriatric

## Abstract

**Background/Objectives:** Osteoporosis is prevalent in the elderly and increases fracture risk. Bone density is commonly assessed using dual-energy X-ray absorptiometry (DEXA). The femoral cortical thickness index (CTI) also provides indirect information for osteoporosis. It remains unclear whether there are intra-individual differences and if a correlation to fracture risk of the CTI in fractured femora results due to fracture related malrotation during X-rays. The aim of this study was to investigate the individual bilateral CTI in patients with proximal femoral fractures. **Methods**: A retrospective analysis of 200 surgically treated patients (100 trochanteric, 100 femoral neck fractures) was performed. Measurements included the bilateral CTI at 10 and 15 cm below the lesser trochanter. Analysis of the correlation of those examinations, in comparison to the contralateral CTI at 15 cm, and correlation of the CTI with the body mass index (BMI) and age was performed. **Results**: Results showed significant differences (*p* < 0.001) in bilateral CTIs for both fracture types at 15 cm with a strong inter-rater reliability (ICC > 0.9). There was no significant correlation between age and CTI, as well as BMI and CTI in both cohorts (*p* > 0.1). Sex-specific subgroup analyses revealed that females exhibited significant differences in CTI between fractured and non-fractured sides (*p* < 0.001). **Conclusions**: In conclusion, CTI, and the modified CTI at 15 cm below the lesser trochanter in fractured proximal femora, is lower compared to the non-fractured side. The femoral CTI could help in daily clinical routines and circumstances, where more detailed risk prediction tools are lacking.

## 1. Introduction

With increasing age, diminishing bone mineral density (BMD) results in osteoporosis. This osseus disease pattern is a condition defined by the World Health Organization (WHO) as a BMD-T-score more than 2.5 standard deviations (SD) below the population mean of young adults, typically assessed via dual-energy X-ray absorptiometry (DEXA) [1,2,3]. Osteoporosis affects around 19.7% of the global population, with higher prevalence in developing countries [4]. As the population ages, the number of affected individuals increases. In the US, the number of over 50-year-old individuals was estimated to reach 71 million by 2030 [5].

This is, continuously, a major issue, as osteoporotic fractures represent the most severe consequence of the disease and pose a major economic burden. In the United States, the annual financial burden exceeds 20 billion USD, with hip fractures accounting for the largest proportion of these costs, primarily due to prolonged hospitalization and rehabilitation requirements [6,7,8]. Hip fractures represent the most severe form of osteoporotic fractures. The majority of hip fractures require hospitalization, and up to 20% of patients die within the first year following the fracture, primarily due to severe underlying medical conditions [9,10]. Hip fractures frequently result in a loss of independence, the necessity of institutionalization, and an elevated risk of subsequent fractures [11].

The risk of fractures due to low-energy trauma and falls increases with age, making osteoporosis a significant burden on healthcare [3,5,12,13,14]. Common osteoporotic fractures include those of the hip, vertebrae, and distal radius [4,12], with case numbers increasing significantly worldwide [12,13,15,16]. These are just some of the reasons why prevention strategies are important.

DEXA measurements remain the gold standard for BMD assessment. However, this measuring modality is not universally available, particularly in emergency or resource-limited settings, and involves additional costs, radiation exposure, and time, whereas X-rays are routine, fast, and low-cost examinations [17,18]. The relationship between various plain radiological parameters and BMD has previously been investigated, including the femoral cortical thickness index (CTI) [19,20,21,22]. The CTI has shown moderate to strong correlation with BMD T-scores and has potential to aid in the identification of local osteoporosis in elderly and postmenopausal populations [21,22,23]. Patient cohorts with femoral fractures were frequently examined, whereas a great heterogeneity regarding the measured side was observed. Previous studies focused on the examination of the fractured femur, which is commonly malrotated in post-traumatic X-rays and, therefore, may lead to distorted measuring results. Until today, it remains uncertain whether there are bilateral intra-individual differences of the CTI in patients suffering a proximal femoral fracture [23,24,25,26]. Measurements of the CTI on post-operative X-rays may be incorrect due to surgical procedures such as endomedullary reaming or implantation of nails, which could potentially modify the cortical thickness [27].

In this retrospective study, the primary objective was to determine whether the contralateral non-fractured femur may serve as a reliable site for estimating bone quality by using the previously established measurement of the CTI. The secondary objective was to evaluate whether CTI measurements correspond with established risk factors for osteoporosis, such as age, sex, or body mass index (BMI). We hypothesized that CTI values differ significantly between non-fractured and fractured femoral bones, and that measurements on the non-fractured side reflect baseline bone status more accurately.

## 2. Materials and Methods

**Figure 1:**
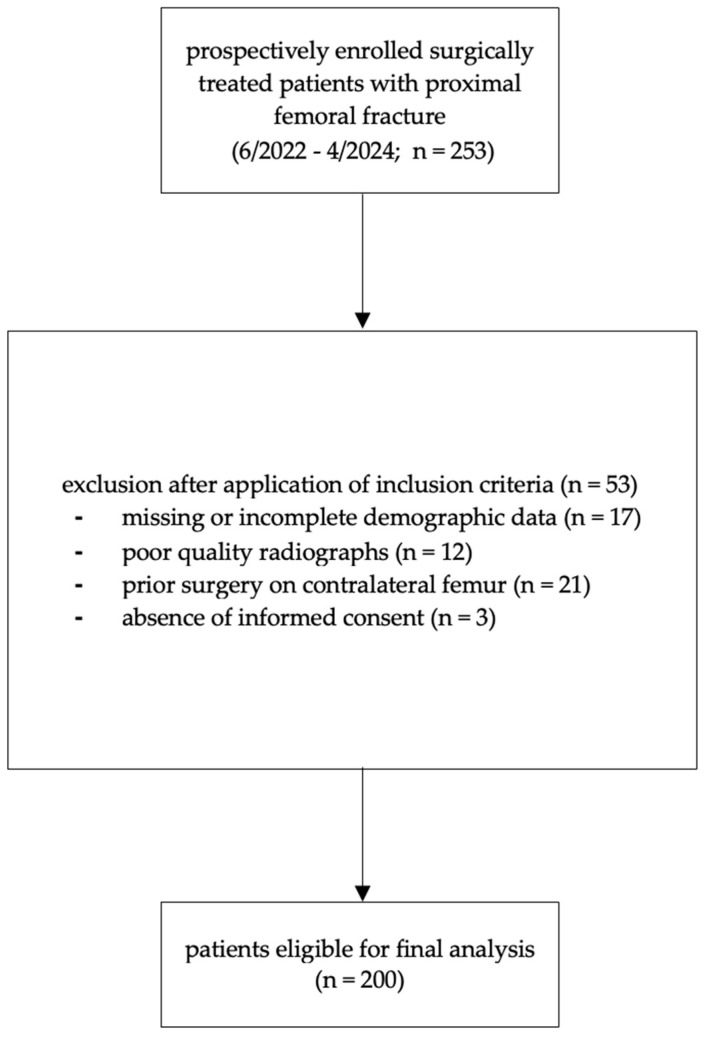
Flowchart of patient selection.

A retrospective analysis was conducted on 200 patients who underwent surgical treatment for proximal femoral fractures (100 trochanteric fractures and 100 femoral neck fractures) between June 2022 and April 2024 at the City Hospital Zurich. Inclusion criteria were as follows: a minimum age of 60 years of age at the time of fracture occurrence, presence of a unilateral proximal femoral fracture (trochanteric or femoral neck), lack of evidence for a pathological fracture, absence of previous surgery on the non-fractured contralateral femur, a complete set of radiographs (pre- and post-operative anteroposterior (ap) pelvis) with correct patient positioning, and the presence of informed consent. Patients not meeting the aforementioned criteria were excluded. Figure 1 shows a flowchart of the patient selection process. Approval of the local ethics committee was given (BASEC number 2024-00290). Patient demographics, previous fractures, and surgical history were extracted from the hospital’s electronic medical record (KISIM, Cistec AG, Zurich, Switzerland). All data were reviewed retrospectively as part of the study design.

### 2.1. Radiographic Measurements

**Figure 2:**
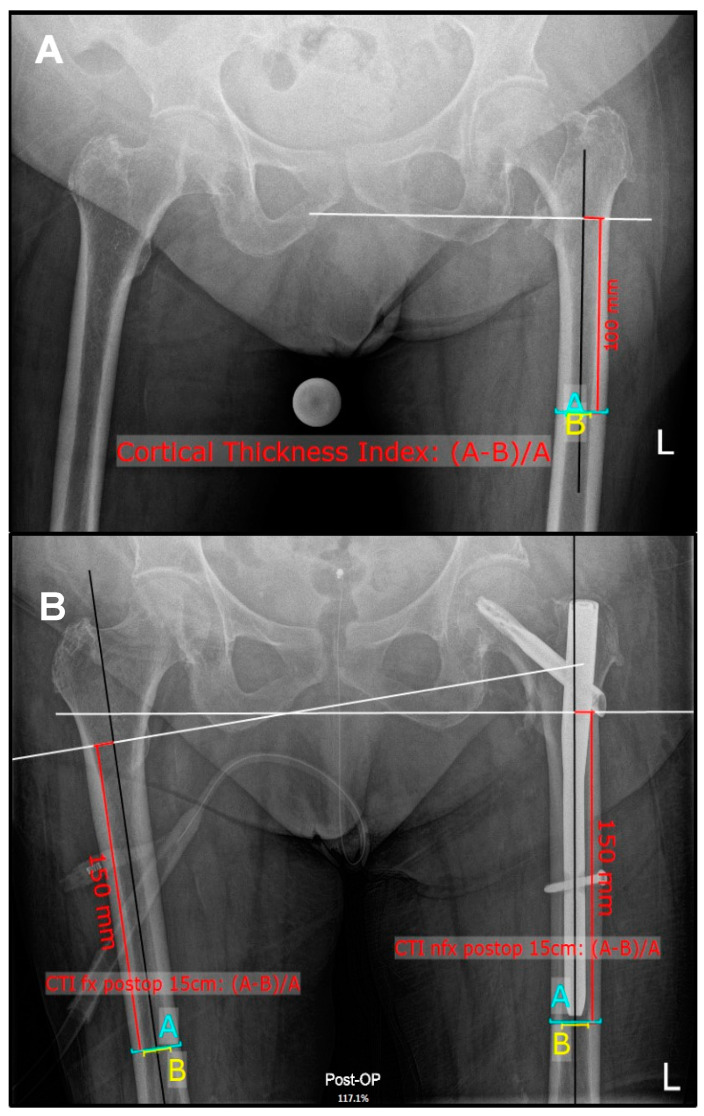
Anteroposterior X-ray of the pelvis: measurement of CTI at ten centimeters below the mid lesser trochanteric line in the left, non-fractured femur (**A**) and 15 cm bilaterally, after implantation of an endomedullary nail (**B**).

The decision was made to solely measure CTI in the anteroposterior projection. As demonstrated in previous studies, ap radiographs have been shown to exhibit higher levels of reproducibility when comparing them with lateral images [26]. In the same study, a significant correlation between the CTI on ap radiographic imaging and BMD assessments were obtained; however, these parameters were not observed in the lateral measurements. The radiology assistant positioned the patient supine with both legs internally rotated (15°) if technically possible. The X-ray was centered on the symphysis pubis. All measurements were performed using mediCAD clinical software Version 6.5 (mediCAD Hectec GmbH, Altdorf, Germany). The outer (A) and inner (B) diameter of the diaphysis ten centimeters below the most prominent point of the lesser trochanter were measured, according to Dorr et al. (Figure 2A) [19], to calculate the CTI (CTI = (A − B)/A). Analysis of the non-fractured contralateral femur was performed on the pre-operative anteroposterior radiograph of the pelvis on the unaffected side for both fracture cohorts. To avoid measurement interference with femoral implants (prosthesis, endomedullary nail) or iatrogenic altered cortical thickness (e.g., through endomedullary reaming) a modified CTI was assessed at 15 cm below the lesser trochanter bilaterally in the post-operative radiograph (Figure 2B). All measurements were carried out by two blinded, independent observers with different levels of experience (orthopedic consultant, medical student) (M.K., F.G.) to assure interobserver reliability. Both observers have previously been trained in measuring the femoral CTI.

### 2.2. Statistical Analysis

An a priori power analysis estimated a number of 100 patients from each group to detect a difference in a small effect size of d = 0.2 using Cohen’s (1988) [28] criteria with a power of 80% and an α of 0.05 using G*Power tool (version 3.1; Universität Düsseldorf Germany). The patients’ clinical features were determined using descriptive statistics. Shapiro–Wilk test was used to evaluate all data for normality distribution. For normally distributed data we used a paired *t*-test, and for non-normally distributed data we used the Wilcoxon signed-rank test to compare the fractured proximal femora with the contralateral uninjured side. To compare continuous variables of the two fracture groups, we used an independent *t*-test (normal distribution) and the Mann–Whitney U test (non-normal distribution). To assess the relationship between radiographic measurements, Pearson’s correlation was used. According to Cohen’s assessment, a correlation of r > 0.5 is considered strong, an r value of 0.5–0.3 is considered moderate, and an r value of r < 0.3 is considered weak [28]. Statistical analysis was carried out using SPSS for Mac (version 23.0, SPSS Inc., Chicago, IL, USA). The level of significance was set at *p* < 0.05. A subgroup analysis with regards to age, gender, and BMI, with those measures being of relevance concerning the change of BMD, was conducted.

## 3. Results

### 3.1. Patient Demographics

For the final analysis, 100 patients of each fracture type (trochanteric, femoral neck) were included. The overall mean age was 84, ranging from 60 to 101 years. The majority of the patients were female (72%) The group-specific distribution of demographic data is shown in Table 1.

### 3.2. Inter-Rater Reliability

The mean inter-rater reliability for the measurements on a height of 15 cm below the lesser trochanter was excellent, with an ICC of >0.9, where an ICC > 0.8 is considered almost perfect and an ICC between 0.8 and 0.6 is considered substantial, according to Landis and Koch [29]. In the group with a trochanteric f fracture, the ICC on the non-fractured side was 0.916 (95%CI: 0.857–0.948), and 0.937 (95%CI: 0.896–0.960) on the fractured side, respectively. For the femoral neck cohort, the ICC on the non-fractured and fractured side was 0.928 (95%CI: 0.869–0.958) and 0.912 (95%CI: 0.848–0.946), respectively. All measurements showed a significance of *p* < 0.001.

### 3.3. Comparison of CTI Within the Subgroups

In both groups, a strong correlation between the CTI at 10 cm and 15 cm below the lesser trochanter was observed (Table 2).

Furthermore, there was a significant difference in the CTI measured 15 cm below the trochanter minor (Table 3).

### 3.4. Correlations Between CTI and Age at Surgery and BMI

When examining the relationship between age and CTI, no statistically significant correlation in both cohorts, on the non-fractured and fractured sides (femoral neck fracture: r = −0.095, *p* = 0.347, resp. r = −0.187, *p* = 0.062; trochanteric fracture: r = −0.151, *p* = 0.134, resp. r = −0.127, *p* = 0.208) were detected. Comparing the BMI with the CTI, no correlations for the fractured and non-fractured sides in the trochanteric fracture (fractured: r = −0.023, *p* = 0.821; non-fractured: r = −0.010, *p* = 926) or femoral neck fracture cohorts (fractured: r = −0.006, *p* = 0.951; non-fractured: r = 0.112, *p* = 0.265) were observed.

### 3.5. Sex-Specific Differences Within the Subgroups

Concerning the subgroup analysis, there was a strong correlation between CTI values at 10 and 15 cm in females in the non-fractured contralateral femur (r > 0.7, *p* < 0.001). When comparing the CTI at 15 cm of the fractured and non-fractured contralateral side, significant differences in all subgroups were found (*p* ≤ 0.001). Males presented an exception, with a femoral neck fracture that demonstrated a trend towards lower CTI on the fractured side (*p* = 0.83). Detailed data can be found in Table 4 and Table 5.

## 4. Discussion

The main finding of the present study is that the CTI 10 centimeters below the mid lesser trochanteric line strongly correlated to the modified CTI at 15 cm in the non-fractured contralateral proximal femora in a cohort of elderly patients with a proximal femoral fracture. Secondly, the modified femoral cortical thickness index fifteen centimeters below the lesser trochanter differs significantly when comparing the fractured side with the non-fractured contralateral femur.

Former studies showed the CTI being significantly lower in patients diagnosed with osteoporosis using DEXA. Additionally, a positive correlation between the CTI and BMD was observed [20,24]. Those correlations were found in patients without a hip fracture. Sah et al. described a positive correlation (r = 0.5814, *p* = 0.0005) between the CTI and T-scores in anteroposterior radiographs for osteoarthritic females [21]. Ilyas et al. observed a strong correlation (r = 0.632, *p* < 0.001) between those two measuring modalities in over 50-year-old females without a hip fracture [22]. Using this knowledge, the CTI and other plain radiological measures of the proximal femur could potentially be a helpful tool to assess the risk for hip fractures [30,31].

However, a certain inhomogeneity regarding the analyzed cohort, or the measurement of CTI and BMD exists. Faundez et al. previously showed a moderate correlation between CTI and BMD in over 45-year-old patients without a hip fracture [25]. Nguyen et al. found the femoral neck BMD to have a significant positive correlation (male r = 0.4, female r = 0.52, *p* < 0.001) with the CTI in anteroposterior radiographs, although they did not observe actual fracture incidence in their study population [23]. In contrast, another study observed the CTI in hip fracture patients to be correlated to the BMD at the femoral neck, finding no correlation to overall BMD [26]. In patients with a unilateral hip fracture, the CTI was found to be helpful in predicting proximal femoral fractures in the contralateral hip [32,33].

Moreover, in various studies, the presence or absence of a fracture was somehow neglected. Several authors measured the CTI in a cohort of non-fractured patients [21,22,25], whereas others observed the CTI in the contralateral uninjured femur of a fracture cohort [26]. Further groups determined the CTI in the contralateral, initially non-fractured side [32,33], and in certain studies it remained unclear on which side the CTI was measured [23,34].

Furthermore, a vast majority of previous studies neglected the fact that fractured femora can be associated with malrotation on radiographs and, therefore, create a different anteroposterior CTI [35,36,37]. Additionally, post-operative radiographs of fractured femora could potentially show altered CTI values due to mechanical medullary preparation (reaming in intramedullary nailing and femoral preparation with rasps in arthroplasty). Those are two potential sources of measuring errors which the present study overcame with the initial comparison of the CTI at 10 and 15 cm below the lesser trochanter, and the secondary measurement of the CTI in post-operative radiographs at 15 cm, where a pain-induced malrotation of the femur was ruled out. The current study shows that the CTI of both sides are significantly different and, therefore, the use of the CTI of the non-fractured contralateral femur to predict fracture risk must be questioned.

Similar to the CTI at 10 centimeters, the CTI at 15 cm showed good to excellent inter-rater reliability. This is consistent with the findings of the good to excellent inter-rater and intra-rater reliability amongst conventional radiological parameters of the proximal femur of previous studies [23,25,26,32,38]. As a reliable method, the CTI could be used as a simple evaluation of patients at risk for a proximal femur fracture as DEXA measuring modality is not universally available, includes additional costs, time, and effort, whereas X-rays are routine examinations [12]. Furthermore, DEXA measurements of the hip have significant limitations for predicting osteoporotic fractures, such as the need for strict quality standards, operator dependency, low resolution, and an inability to directly image bone microstructure [20,39,40].

Looking at the subgroup analyses for both sex-specific differences, the two CTI values at a height of 15 and 10 centimeters on the non-fractured contralateral side strongly correlated with each other. Therefore, a comparison of the CTI at 15 cm within both fracture groups with that of the respective opposite side was performed. A significant difference was found in most groups, except for males with a femoral neck fracture, likely due to the small sample size.

Moreover, no correlation between age and CTI was shown, for both the fractured and the non-fractured side in both cohorts. This issue might have been due to the CTI not being informative enough to show inverse correlations between BMD and age, as other studies have found in the past with DEXA measurements [20]. Again, other studies in the past have found correlations between age and cortical thickness, although their results focused on the femoral shaft or distal femur, and other measurement methods to examine cortical thickness were used [41,42].

The current results regarding BMI and CTI were not as expected, mostly showing insignificant weak inverse correlations, while other studies have found a positive correlation between BMI and cortical thickness [43,44]. This issue might have been due to the current smaller sample size, or measurement angle of the CTI, as there is no literature yet about CTI measurements at a height of 15 cm distal to the mid lesser trochanteric line.

This study has certain limitations: besides its retrospective design, the study contained a rather small sample size. Furthermore, no comparison of BMD measured by DEXA and CTI was conducted. Moreover, a comparison with a cohort of non-fractured patients was lacking. However, a recent study showed that the CTI of non-affected individuals did not show intra-individual differences when comparing both femora [38].

## 5. Conclusions

In conclusion, the CTI and modified CTI at 15 cm below the lesser trochanter in the fractured proximal femora are lower compared to the non-fractured contralateral side. The femoral CTI could help in daily clinical routine and circumstances, where more detailed risk prediction tools are lacking, in order to predict fracture risk in elderly patients. Furthermore, it may be used for the detection of local osteoporosis.

## Figures and Tables

**Table 1:** Patients’ demographics characteristics.

Variable	Femoral Neck Fracture	Trochanteric Fracture
Number of patients	100	100
Sex, n (%)		
female	70 (70)	74 (74)
male	30 (30)	26 (26)
Age at time of surgery, years ± SD	82 ± 9	85 ± 8.45
BMI (kg/m^2^) ± SD	23.3 ± 4.29	22.9 ± 4.15
Body weight (kg) ± SD	64.3 ± 12.46	61.7 ± 12.33
Affected side, n		
left	41	50
right	59	50

SD = standard deviation.

**Table 2:** Correlation of CTI at 10 and 15 cm below lesser trochanter on the unaffected side.

	CTI ± SD NFx at 10 cm	CTI ± SD NFx at 15 cm	r Value	*p* Value
Femoral neck fracture	0.544 ± 0.061	0.502 ± 0.06	r = 0.664	*p* < 0.001
Trochanteric fracture	0.512 ± 0.078	0.481 ± 0.072	r = 0.766	*p* < 0.001

CTI = cortical thickness index, SD = standard deviation, NFx = non-fractured. Data are presented as mean ± SD, significance was set at *p* < 0.05.

**Table 3:** CTI values at 15 cm on both sides, as well as the significant differences for both groups.

	CTI ± SD NFx at 15 cm	CTI ± SD Fx at 15 cm	*p* Value
Femoral neck fracture	0.502 ± 0.06	0.478 ± 0.065	*p* < 0.001
Trochanteric fracture	0.481 ± 0.072	0.457 ± 0.069	*p* < 0.001

CTI = cortical thickness index, SD = standard deviation, NFx = non-fractured, Fx = fractured. Data are presented as mean ± SD, significance was set at *p* < 0.05.

**Table 4:** Correlations comparing the CTI values in the non-fractured side of the subgroups.

Variable	Femoral Neck Fracture Females	Femoral Neck Fracture Males	Trochanteric Fracture Females	Trochanteric Fracture Males
CTI ± SD NFx at 10 cm	0.540 ± 0.062	0.552 ± 0.057	0.514 ± 0.079	0.506 ± 0.077
CTI ± SD NFx at 15 cm	0.488 ± 0.064	0.534 ± 0.045	0.473 ± 0.075	0.504 ± 0.069
Pearson’s correlation	r = 0.716	r = 0.538	r = 0.807	r = 0.742
*p* value	*p* < 0.001	*p* = 0.046	*p* < 0.001	*p* = 0.841

CTI = cortical thickness index, SD = standard deviation, NFx = non-fractured. Data are presented as mean ± SD, significance was set at *p* < 0.05.

**Table 5:** Significant differences comparing the CTI values of the fractured and the non-fractured side.

Variable	Femoral Neck Fracture Females	Femoral Neck Fracture Males	Trochanteric Fracture Females	Trochanteric Fracture Males
CTI ± SD Fx at 15 cm	0.461 ± 0.06	0.517 ± 0.062	0.451 ± 0.072	0.474 ± 0.058
CTI ± SD NFx at 15 cm	0.488 ± 0.064	0.534 ± 0.045	0.473 ± 0.075	0.504 ± 0.069
*p* value	*p* < 0.001	*p* = 0.083	*p* < 0.001	*p* = 0.001

CTI = cortical thickness index, SD = standard deviation, Fx = fractured, NFx = non-fractured. Data are presented as mean ± SD, significance was set at *p* < 0.05.

## Data Availability

The datasets presented in this article are not readily available because of ongoing studies. Requests to access the datasets should be directed to the corresponding author.
